# Wireless Energy Transfer Powered Wireless Sensor Node for Green IoT: Design, Implementation and Evaluation †

**DOI:** 10.3390/s19010090

**Published:** 2018-12-28

**Authors:** Janne Janhunen, Konstantin Mikhaylov, Juha Petäjäjärvi, Marko Sonkki

**Affiliations:** 1 Solmu Technologies, 90620 Oulu, Finland; janne.janhunen@oulu.fi or janne.janhunen@solmutech.com (J.J.); juha.petajajarvi@oulu.fi or juha.petajajarvi@solmutech.com (J.P.); 2 Centre for Wireless Communications, University of Oulu, 90570 Oulu, Finland; marko.sonkki@oulu.fi

**Keywords:** wireless power transfer, radio frequency, energy harvesting, low power technology, green IoT, application, methodology

## Abstract

The number of IoT (Internet of Things) devices is predicted to increase dramatically in the years to come and their manufacturing and maintenance, including both commercial and ecological aspects associated with these, are gaining substantial attention. One of the effective ways of addressing both these issues at a time is the energy-neutral systems, which operate with the energy harvested from their environment. To address the major problem of this system, namely the low reliability, in the current paper, we develop and study the utility of a system powered solely with the wireless power transfer (WPT) over a radio frequency (RF) channel. In the article, we propose a methodology for developing and implementing a real-life IoT application based on RF WPT. We employ the proposed methodology to develop a WPT-powered solution to sense the temperature and the angular velocity in the rotating industrial environment. First, we discuss the key trade-offs arising when selecting and developing the new components for a WPT system. Then, we present and detail our solutions and describe the results of their evaluations. Finally, we instrument and evaluate the complete system, proving that it is capable of meeting all the design goals and requirements. The results reported in this paper can be of interest to the practitioners, for whom they provide a step-by-step methodology of WPT application development with a practical example. In addition, these results may be valuable for analysts, as they demonstrate many practical interrelations and effects specific to the real-life WPT applications.

## 1. Introduction

Millions of new sensing devices have been deployed in recent years, and many more will take their places in the years to come. This makes a decisive step towards transforming the sparse IoT (Internet of Things) of today into the massive IoT. The IoT devices are employed for an extensive range of applications—from military to health and well-being monitoring, from environment sensing to vehicle tracking [1]. Despite all the difference in the requirements and the key performance indicators originating from the very nature of these various use cases, the structure of the majority of the sensing devices, called nodes, is today rather uniform. Namely, a typical commercial IoT node is composed [2] of a microcontroller, controlling its operation, a radio transceiver, providing means for communication, the set of application-specific peripherals (typically—sensors and actuators) and an energy source, typically represented by a primary or a secondary battery, or mains. The devices like these have been in use already for many years and have shown themselves well.

Nonetheless, the limited lifetime of such devices still represents one of the major limitations for such systems. Luckily, the recent advances in respect to the ultra-low power signal processing and wireless communications provide means to approach this challenge by replacing a battery with a solution enabling a node to get powered with the energy available around it. This enables for the battery-free sensor applications, which are more ecologically friendly and can enjoy more simple service and maintenance.

To give an example, the nodes in a sensor network feature a self-forming infrastructure, developing a configuration based on the application as well as on the environment [3]. The capability of communicating over wireless channels and without batteries allows deployment in harsh and very dynamic environments—for instance, in smart mechanical components inside an engine where vibrations and moving objects would abstain from supplying power over cables or placing the sensors with heavy and volatile batteries. Another motivation for getting rid of the cells is due to safety and ecological constraints. The heat-up of the moving parts along with the possibility of suffering mechanical damage obstruct the use of batteries, which include flammable or hazardous materials. Finally, removing the cells for extremely large numbered sensor networks may bring substantial savings. Therefore, with energy harvesting, the applications in which using sensors was impossible or too expensive now become a reality.

It is evident that, to enable energy transmission or harvesting of the energy, the consumption of a node should be balanced with the available energy income, thus implementing an *energy neutral* system [4]. This inevitably requires minimizing the energy consumption of all three critical functions of a sensor node: processing, sensing, and communication. The two former ones are heavily dependent on the features of the hardware (HW) components used, which, in their turn, are dependent on the requirements of a particular application scenario. To give a practical example, the contemporary digital-output temperature and humidity sensors typically consume under one milliWatt and have millisecond-order response time (e.g., [5,6]). Meanwhile, the accurate carbon dioxide sensors feature hundred milliWatt order consumptions with response times of up to ten minutes [7,8]. The processing system should be selected while mindful of the complexity of the operation algorithms and the respective memory requirements. In addition, one needs to keep in mind the extra energy cost for keeping up the non-volatile memory content, and the difference in the energy efficiency of computing for general processors and application-specific co-processors and accelerators.

Finally, the wireless communication can also be implemented in many various ways [2,9,10]: employing the short range (e.g., IEEE 802.15.4 or Bluetooth Low Energy (BLE)) or the long range (e.g., Low Power Wide Area Network (LPWAN) technologies), operating in the license-free Industrial, Scientific and Medical (ISM) or in licensed (e.g., Long Term Evolution (LTE) or the recently introduced cellular IoT technologies) frequency bands, etc. The short-range technologies typically outperform the other alternatives with respect to their consumption due to the lower transmit power, the short on-air time, and the more simple protocols. The downside of these systems is the need for more dense infrastructure. The long-range technologies, and particularly, LPWAN systems, typically feature much higher consumption and thus require a node to process and store much more energy for communication, which increases the complexity and cost of the node.

There are several alternatives from where a node may attempt to obtain energy for its operation, including, e.g., the solar light, vibration, etc. Nonetheless, most of the potential energy sources are ambient and thus very opportunistic. In contrast to them is the wireless energy transmission, which implies purposeful delivery of the energy to a device in some form over a wireless channel. The comprehensive survey of wireless energy transmission techniques and technologies has been presented in Ref. [11] and references therein. The typical wireless power transfer (WPT) system consists of the three components—the base station emitting the signal carrying the energy, the transmission channel and the energy harvester. The latter typically represents a system receiving the signal (e.g., an antenna) and converting the high-frequency signal to the direct current (DC) (i.e., the rectifier). The survey presents various energy-harvesting circuit topologies and state-of-the-art Schottky diode based circuit designs. The obtained efficiencies range from 1.2 to 72.8 percent in 869–2450 MHz frequency range for input radio power levels of −25 to over +60 dBm [11]. Even though the losses are still quite high, they may already enable particular real-life IoT applications.

Therefore, in this study, we propose a methodology for designing a real-life IoT application based on radio frequency (RF) wireless power transfer. Then, we show how this methodology can be employed by developing a WPT-powered solution to sense the temperature and the angular velocity in the rotating industrial environment. These two constitute the main contributions of this paper. Importantly, in the process of our development, we discuss the key trade-offs arising when selecting and designing the critical components of the system: the sensor node, the RF energy harvester, the antennas, and the WPT transmitter. For each of these components, we present and detail our solutions and describe the results of their evaluations. Finally, we integrate all the developed elements and evaluate the complete system, proving that it is capable of meeting all the design goals and requirements. To the best of our knowledge, this is one of the first attempts to propose a methodology of engineering a WPT-based solution, and to develop the complete real-life IoT application based on the non-inductive WPT concept. The very initial results of this study have been reported in [12].

The rest of the paper is organized as follows. Section 2 presents and discusses the related works. In Section 3, we first introduce our proposed development methodology for the WPT-powered applications. Then, we detail our target scenario and show how our proposed methodology can be applied to develop the practical IoT application based on RF-based WPT power supply. The evaluation results for individual components of our solution and the system as a whole are reported also in Section 3. Section 4 concludes the paper, summarizes the results and points out the further challenges and the directions of future works.

## 2. Background and Related Works

The radio frequency identification (RFID) is currently the most widely used real-life technology, which utilizes the RF-based WPT principle. The transmitted by the reader radio signal is received, remodulated and backscattered by a generally-passive tag. The traditional RFID tags were sending only the static information, stored in their non-volatile memory: the unique identifiers, serial number or product-related data. Nonetheless, the contemporary tags can integrate one or multiple sensors ([13,14,15]), which are powered from the incoming signal and data of which are scattered back. The two principal limitations of the RFID technique are the very limited complexity of the respective applications due to the scarce power budget availability, and the need of having a reader (or the transmitter) device decently close to the tags for the system to run.

The ways to overcome these limitations have been proposed in several studies. For example, in Ref. [16], the authors introduced into a tag a designated energy harvester combined with an energy buffer. The collected and accumulated energy was used to feed the power amplifier, thus extending the potential range of communications. Among other notable approaches, enabling to extend the functionality of RFID, are the bistatic and ambient backscatter approaches (refer, e.g., to Ref. [17]). The former implies that the RF signal source and the receiver of the backscattered signal are different devices. This approach enables for simplifying and optimizing the structure, thus enabling for performance improvements and cost optimizations. The latter approach refers to tags using the signals from ambient RF sources (e.g., television or radio, or WiFi) rather than that originating from the dedicated RF sources.

Albeit these modifications improve the utility of RFID dramatically, their functionality remains limited and cannot match that of the conventional IoT devices. The aspects related to engineering and employing RF WPT to power the IoT devices were investigated in a series of studies. The thoughtful survey of the device-level aspects is provided in Ref. [11]. To get an insight into the potential performance of the WPT solutions, in Table 1, we overview the efficiencies of some prototype WPT implementations reported in the literature. Note that Table 1 illustrates the efficiencies for RF–DC conversion and thus does not account for the power losses in the radio channel.

Aside from the research prototypes, the RF-based WPT commercial solutions are also available. For example, the P2110B RF energy harvesting chipset [18] from Powercast^®^ (Pittsburgh, PA, USA) can collect energy from the signals of above −14 dBm and features RF–DC conversion efficiency of about 62% in 900 MHz band for three dBm input signal. The major downside of this solution is the cost—which exceeds 30 EUR (34 USD) per a single receiver for the small-scale orders.

A significant percentage of the electromagnetic signal energy is lost when the signal travels through the radio channel—this is the law of nature. The losses can be somewhat mitigated by operating in the line-of-sight and directing the power transfer towards the receiver. Choi et al. [39] proposed an adaptive multi-antenna system with channel estimation, duty-cycle control, and beamforming algorithms, which dynamically steer the beams towards the sensor nodes. Experimental results indicate that the beamforming allows for increasing the amount of received power up to six times compared to the random beamforming scheme. Another promising observation is that the RF energy transfer efficiency linearly increases with the number of transmit antennas when the beamforming algorithms are applied. In Ref. [40], Choi et al. extended the previous research by combining beamforming and energy neutral control algorithm. The techniques balance the transferred power and the consumed power, i.e., the power beacon provides more energy towards the node with low energy in its buffer, while the node reduces power consumption by lowering its duty cycle. In Ref. [41], the same authors continued to study the phase and frequency synchronization problems in a distributed wireless power transfer system. Although the distributed system comprises several technical challenges, the results show that the coverage problem related to the wireless power transfer can be improved applying distributed system.

Finally, the presence of the WPT needs to be also accounted for at the network level. A good overview of the respective challenges and potential solutions is provided in Ref. [42]. To this day, the problems of optimizing data and energy transfer (within simultaneous wireless information and power transfer (SWIPT) concept) [43,44,45], the beam width and direction [46,47], design of media access and routing [48,49,50], interference management [51,52] and many others have been addressed analytically and via network-level simulations.

Despite all these immense progress in respect to the individual components, the number of the complete IoT solutions employing WPT is still very scarce today. The primary reason for this is the complexity of balancing all the critical requirements for such systems, i.e., the energy efficiency (1) both in transmission and conversions, small form-factor (2), and low-cost (3), within the power and duty cycle (4) regulations of today. Therefore, in the following sections, we investigate how one can design a real-life IoT application based on the RF-based WPT principle. We start by discussing the methodology of designing a WPT-enabled device. Then, we proceed with justifying and describing the employed technical solutions. Finally, we present the results of the evaluation and discuss the lessons learned.

## 3. Development of RF WPT-Enabled IoT Application

### 3.1. Design Methodology

**Figure 1:**
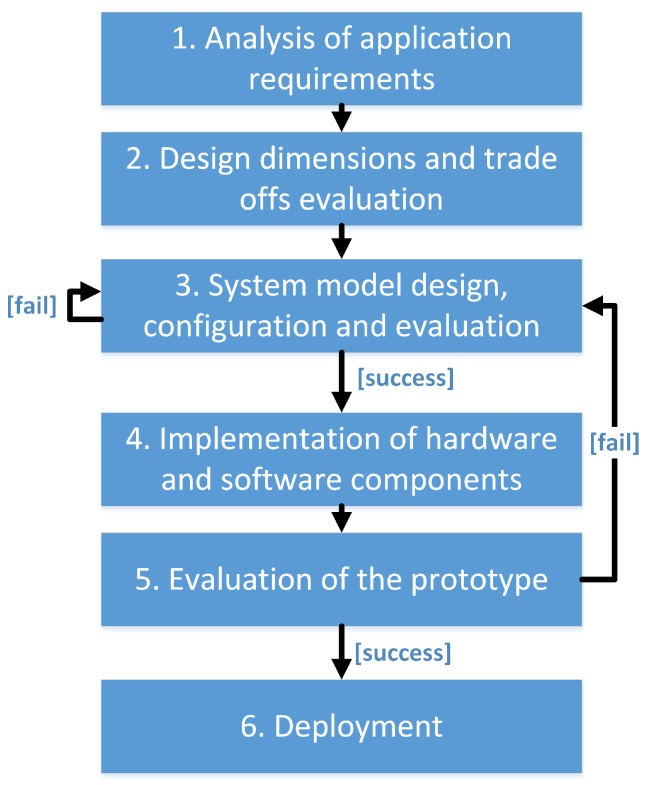
Traditional design flow for a wireless sensor node (based on methodology presented in Ref. [54]).

The general methodology for the development of embedded applications and specifically its application towards the design of the wireless sensor network (WSN) solutions have been discussed in [53,54], respectively. The traditional design flow consists of six phases, depicted in Figure 1 [54]:

1. In the first phase, the application is analyzed, and the requirements, desired functionalities, and operating environment get identified.
2. The data collected during the first stage are used for evaluating the design dimensions and balancing the respective trade-offs during the second stage.
3. The defined design dimensions and functionalities become the input of the third phase. During this phase, the technical requirements are defined, and the system’s architecture, as well as the architectures of the HW and software (SW) components, are chosen. The completion of phase three may require multiple iterations or even result in a return to phase two if it is found that the defined requirements cannot be fulfilled.
4. The focus of the fourth phase is on the design and implementation of the new HW and SW components, configuration or modification of the already-existing components and the integration and testing of all the components as a single system.
5. Once all the components of the system are implemented and tested, a few nodes are prototyped and deployed in the test environment to test the networking mechanisms and check the performance of the network as a whole. If some problems are detected, the design may return to one of the earlier phases.
6. If no problems are detected, the application is deployed and tested on a large scale, and, if it succeeds, it may enter mass production.

When this comes to the energy harvesting powered applications, these general guidelines and specifically phases 1–3 need to be adapted. Unfortunately, we are not aware of any previous attempts to methodologize the procedure of developing and in what follows we address this obvious omission.

The principal difference between designing a conventional WSN application and the one, to be powered with energy harvesting, is the significance and depth of accounting the environment and its effects. For the conventional battery-powered system, the environment primarily affects the operation of the sensors (e.g., consider the sun heating up a sensor, making it report wrong pressure value) or change the radio channel condition (e.g., consider a car blocking the line of sight between a sensor node and a gateway). Meanwhile, for the systems based on energy harvesting, the changes in the environment (e.g., the closed curtains on a window) may drastically change the amount of energy available to the sensor nodes.

Therefore, when analyzing the requirements of an EH-based application, the special attention needs to be paid to the study of the environment of operation and the dynamic processes happening there. This study must specify what orders of energy can be harvested in this particular environment from different sources. What is more important is that this study should also come up with the *energy availability model*, which would answer such questions as:

- Does the energy become available periodically or randomly?
- How long can the period of energy unavailability last?
- How big can be the fluctuations in the energy?
- Are there any particular trends in energy availability patterns?
- How does the collected energy scales up depending on the position and design dimensions of the device?

In parallel with the energy availability model definition, the model for IoT device consumption also needs to be determined. The careful comparison of these two models shows whether the target application is feasible and what are the key design dimensions and trade-offs. Specifically, these analyses determine such important design factors as the general system architecture and protocols, the used energy harvesting mechanism and the requirements to the harvester (e.g., size, weight, efficiency), the need of using an energy buffer and its volume. Following these steps, the development of the system following the standard procedure can commence.

Unlike the opportunistic energy harvesting based applications discussed above, the availability of energy in the RF WPT-based applications has a more predictable nature. Given that the schedule of WPT charger is known, the power of the radio signal received by an IoT device depends on the distance to the charger, the antennas used and the radio channel conditions (e.g., channel blocking). At the very same time, the WPT introduces a new design challenge, which is the development of WPT transmitter and positioning of the WPT infrastructure.

Specifically, based on the analysis of the target functionalities and the operation environment, one needs to determine whether RF WPT-based supply is technically and economically feasible. Next, the number and the location of the WPT chargers, the frequency of operation, the shape of the WPT radio signal and the antenna polarization need to be determined. Finally, all respective components need to be designed, instrumented and evaluated in real life. In what follows, we illustrate step-by-step how this methodology can be employed to develop an illustrative WPT application.

### 3.2. Target Application and Requirements

As a target scenario for our illustrative WPT implementation, we selected a rather common industrial use case coming from the field of mechanical engineering, which is the measurement of the acceleration of the rotating mechanical structures. The need for such a solution arises in production (e.g., consider a metalworking lathe [55,56]), power generation (e.g., generator turbines [57]), transportation (e.g., the ship or aircraft screw propeller or propeller shaft—[58]) and many other domains. Even the conventional cars may benefit from having a reliable system monitoring the parameters of their wheels [59].

This is obvious that a cable connection to the sensors located on a rotating structure to read their data and provide them the power is hardly feasible. Therefore, up to now, two different approaches were primarily employed to address this challenge. The former one bases on the use of static sensors located around the rotating structure (e.g., cameras, microphones, magnetic or vibration sensors), the data from which are analyzed to determine the parameters of interest. Another option is mounting a battery-powered sensor to a rotation structure directly. In what follows, we attempt to address the very same challenge in a new way, employing the RF-based WPT.

The key requirements for our use case are as follows. Given a lathe with a disc with a diameter of 30 cm, rotating around the horizontal axis with the speed of 60 rpm. The lathe is open from one side, providing a line-of-sight towards the rotating disc. A battery-free sensor device constantly measuring and reporting over a wireless channel at least twice per second the temperature and the acceleration must be mounted on the disc. The sensor should be fully powered with RF-based WPT, with WPT transmitter located at a distance of at least one meter from it.

### 3.3. Feasibility Analysis and Design Dimensions Definition

To analyze whether the target application is feasible, we start with a brief analysis of the application’s energy utility. The state-of-the-art accelerometers (e.g., LIS2DE12TR from STMicroelectronics (Geneva, Switzerland) or BMA253 from Bosch Sensortec (Kusterdingen, Germany) consume about 30–300 μW in average when active, depending on the sampling rate. The temperature sensors (e.g., SI7053-A20-IM from Silicon Labs (Austin, TX, USA)—below 10 μW in average for one sample per second (sps) measurement. The consumption of a system-on-chip composing a BLE transceiver and a microcontroller (e.g., CC2650 from Texas Instruments (Dallas, TX, USA)) is on the order of 60 mW while transmitting with the microcontroller active (with the on-air time of below 2 ms) and below 30 μW in idle. Therefore, the expected mean power consumption is in the range of 200–500 μW. Note that, to simplify the analysis of the applicability of energy harvesting and WPT for other applications and scenarios, we provide Table 2, capturing the consumption of the state-of-the-art commercial sensors.

Next, we consider the amount of energy, which one can deliver to the node over WPT. For this, we start by analyzing the frequency regulations. Since the system will be deployed in Europe, we refer to the documents [60,61]. Note that, to the best of our knowledge, none of the regulatory agencies to this day have issued specific regulations for RF-based WPT systems. For this reason, we consider the regulations for the non-specific short-range devices operating in ISM bands, which are summarized in Table 3.

**Figure 2:**
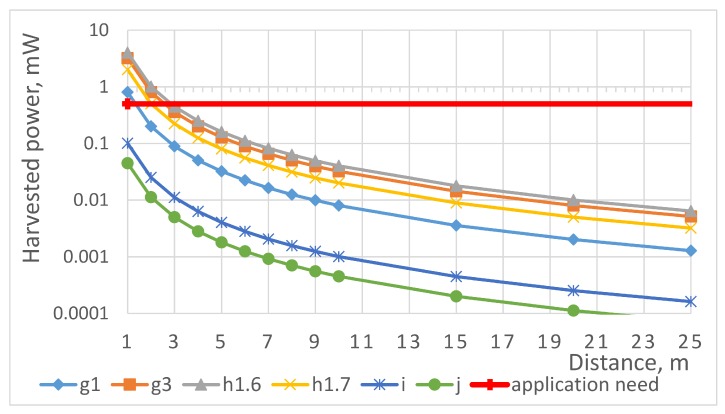
Maximum power transferred via wireless power transfer in different Industrial, Scientific and Medical bands (assuming use of dipole antennas and 50% radio frequency-direct current conversion efficiency.

Next, we assume that both WPT transceiver and receiver antennas are omnidirectional (e.g., dipoles with 2.15 dBi gain) and that the efficiency of RF–DC conversion is 50% (refer to Table 1). In addition, we use the free-space propagation model to account for the losses in the channel. Consider that the RF energy is sent in 2.4 GHz frequency band with the maximum Effective Isotropic Radiated Power (EIRP) of 10 mW. At one meter distance, the propagation loss is in the order of 37 dB and the total harvested power is in the order of 100 μW, which is below the level needed by our targeted application. Next, we consider the h1.6 band featuring 500 mW effective radiated power and 10% duty cycle limitation. At a distance of about 2.8 m, the harvested power exceeds 0.5 mW, thus enabling supplying the target device. Finally, for band g3, we can estimate that the target level of 500 μW is met at a distance of about 2.5 m. The calculated maximum levels of power transferable via WPT for the different ISM bands accounting for the duty cycle restrictions are summarized in Figure 2.

As one can see from Figure 2, among all the ISM bands available, the h1.6 band enables transferring the maximum amount of energy. In addition, the presented results reveal that the amount of energy available exceeds the amount required for powering our target application. Therefore, we can conclude that the targeted application is feasible.

Note that the previous analysis implies the use of omnidirectional antennas and the match of polarization between the WPT transfer and receive antennas. Meanwhile, for the targeted use case, this is also possible to employ directional antennas, thus reducing the power losses in the radio channel. At the same time, in the case, if antennas are not circularly polarized, the rotation may result in energy losses due to polarization mismatch. These two effects also need to be investigated experimentally in the process of design.

### 3.4. Implementation and Experimental Validation

The presented above analysis showed that the target application is potentially feasible and provided valuable insights into its implementation (e.g., the band). In the current section, we will go step-by-step through the key components of the system, justifying their selection or describing their design and evaluation.

#### 3.4.1. Selection of a Sensor Node

This is obvious that the very low energy consumption of the IoT sensor node is crucial for the success of the targeted application. Today, there are many IoT sensor solutions available on the market, including the ones designed for energy-harvesting supply. Therefore, we start our development by selecting the appropriate sensor node device, which has to address the following challenges.

The first challenge is related to the possibility of strong variation of the input radio signal’s power resulting from, e.g., the mobility of the device, blockage of the radio channel, or the temporal pattern of the base station operation. To address this challenge, the power system of the IoT device needs to combine a DC–DC converter stabilizing the supply voltage as well as an energy buffer, which would provide the energy when no radio signal is available or when it is too weak.

Notably, the peripheral components (e.g., sensors and actuators) of an IoT node may require possessing different voltage levels. In this case, the feasibility of using such components and of generating multiple supply voltage levels need to be carefully evaluated, since each new DC voltage increases the total energy consumption, especially in the case if the desired voltage is higher than the original one. At the very same time, the use of higher supply voltages may increase the power consumption, which is especially undesirable for energy-harvesting powered systems. Therefore, a single-voltage DC–DC converter generating the minimum level of the voltage needed for the operation of the whole IoT node is typically the most efficient solution.

The energy buffer, in its turn, must store sufficient amount of energy to support the operation of the node during the period when no energy income is available. Depending on the use case and the environment of operation, as an energy buffer can serve a conventional capacitor, a supercapacitor or a secondary battery. The former is characterized by small capacity and thus can only help to handle decently short (typically—minute-order) periods of energy unavailability, but are less expensive than the other options. The supercapacitors have higher capacitance and thus can enable longer autonomy, but are much more costly. Finally, the secondary batteries feature decently high capacity and reasonable costs, but often need high current to get effectively charged.

The key requirement for the processing and the wireless communication systems is the low consumption not only in sleep mode but also in active mode. The radio technology needs to feature low peak and mean power consumptions, which calls for the technologies with low transmit power, high over-the-air rate, and simple communication protocols. The BLE or IEEE 802.15.4 are typical examples of such radio technologies. Another important aspect is the reduction of consumption for communication between the processing core and the radio. In this respect, the system-on-chip based systems outperform the ones with discrete microcontroller and radio.

**Figure 3:**
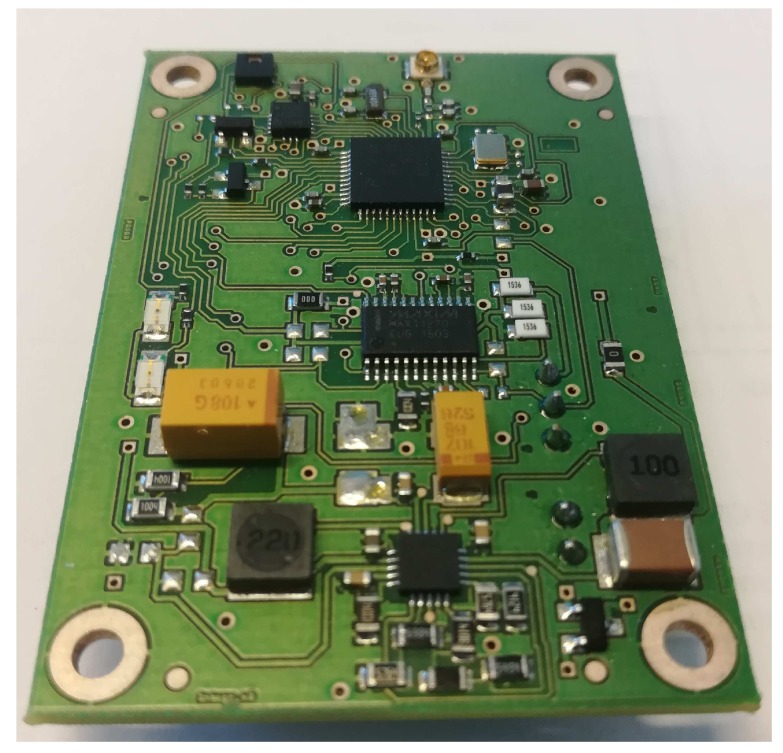
An integrated sensor node by Solmu Technologies.

**Figure 4:**
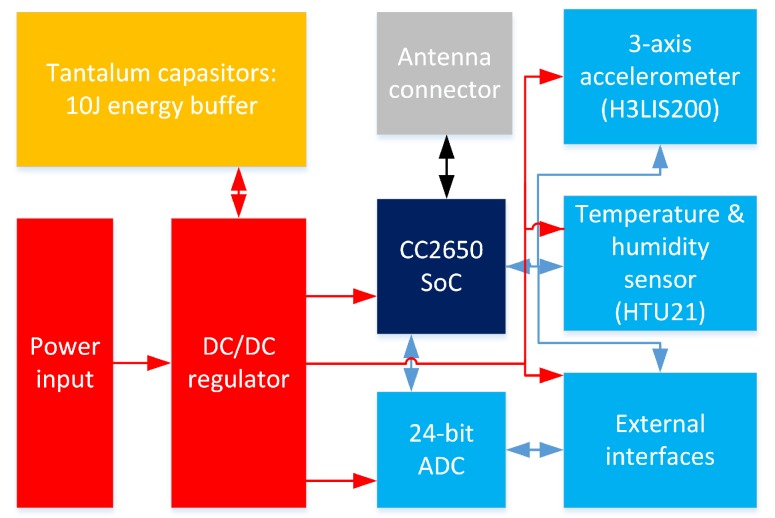
Structure of the sensor node.

Finally, the proper selection of the sensors and other peripherals is of extreme importance. Based on the discussion above, for our implementation, we selected a sensor node from Solmu Technologies (Oulu, Finland) [62] designed and optimized specifically for energy-harvesting powered operation. A node (depicted in Figure 3, the structure is shown in Figure 4) has in its core a multi-radio-technology-enabled system-on-chip based on 32-bit Advanced RISC Machine (ARM) controller (consuming 100 nA in shut down and below 3 mA operating with a clock of 40 MHz). To enable the energy harvesting supply, the node is equipped with a DC–DC converter, which can convert an input voltage from 0.1 V to over 5 V to a stable DC voltage in the range from 2 to 3.6 V. Importantly, having the ten μA current on its input, the converter demonstrates the efficiency of over 80%. As the energy buffers, the tantalum capacitors storing up to 10 J of energy storage in total are used. A sensor node also has the place for a single CR2032 lithium battery, which can be used as an alternative or a backup power source option. The node is equipped with an instrumental-grade external analog-to-digital converter and with low-consuming environment sensor (i.e., temperature and humidity) and three-axis acceleration sensors.

**Figure 5:**
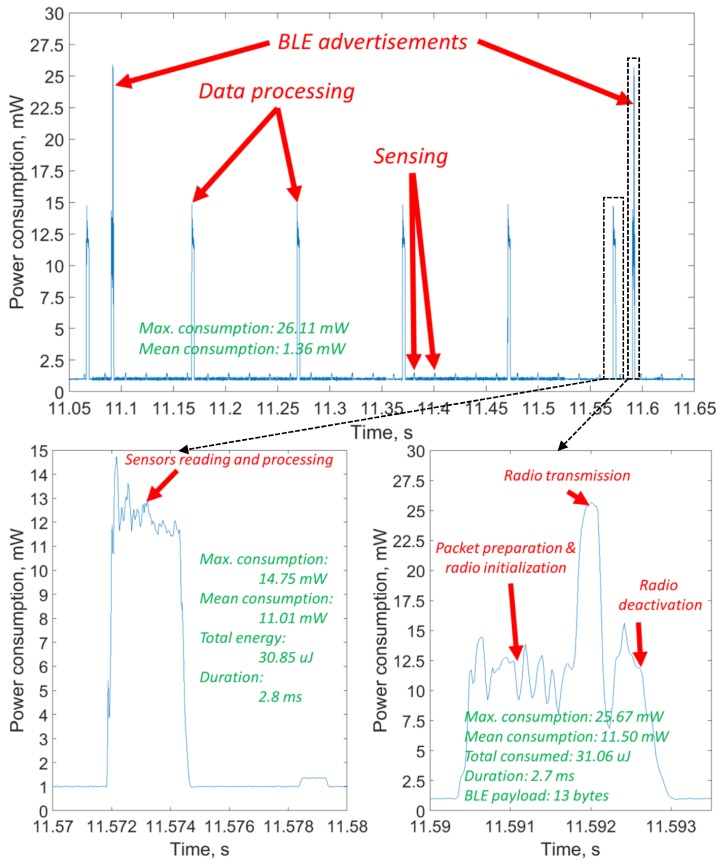
Measured power consumption profile of the sensor node.

For this node, we have developed the firmware, making the sensor node measure the acceleration, process it (i.e., calculate the Fast Fourier transform (FFT) to estimate the speed of rotation) and the temperature, and send them twice a second in BLE advertisements. The practical energy consumption profile of the node for this specific application, which was measured with an Agilent N6705 power analyzer (Santa Clara, CA, USA) and then visualized in MATLAB, is depicted in Figure 5. The data processing and communication phases, and the key numbers related to the consumption are shown in the picture. One can see that the mean power required for stable operation of the system is 1.36 mW.

This consumption is higher than the one we expected and used in our feasibility analysis. The three reasons for it are the higher consumption of the sensors, the extra consumption for preparing the packets for the BLE, and, especially notable, the additional consumption for signal processing. The calculation of the FFT required quite substantial processing, which also brought along with it the extra consumption. Nonetheless, one can see that, even with this consumption, the application stays feasible for WPT in h1.6, even though the communication range will likely decrease to below 1.5 m.

#### 3.4.2. Antennas for RF Energy Transfer

The antennas of both the base station and the IoT node are the key elements, affecting the efficiency of the WPT. Depending on the number of the nodes served by a single WPT transmitter and whether the location of all the IoT nodes is known and static, one can use two different approaches. The former one is based on the use of omnidirectional antennas and is intended for the case when the nodes are uniformly scattered around the base station or are highly mobile. As analyzed, e.g., in Ref. [46], this approach is characterized by the minimum energy transfer efficiency. The use of directive antennas enables increasing the WPT efficiency (refer, e.g., to Ref. [63]) but is sensible only if the location of the nodes is known or if they are clustered most of the time. Finally, a combination of these techniques (e.g., use of directive antennas for base station and omnidirectional on the nodes, or vice versa) can be considered for particular use cases. Note that the requirements of the real-life applications often limit the antenna dimensions, thus putting a limit on antenna’s efficiency.

**Figure 6:**
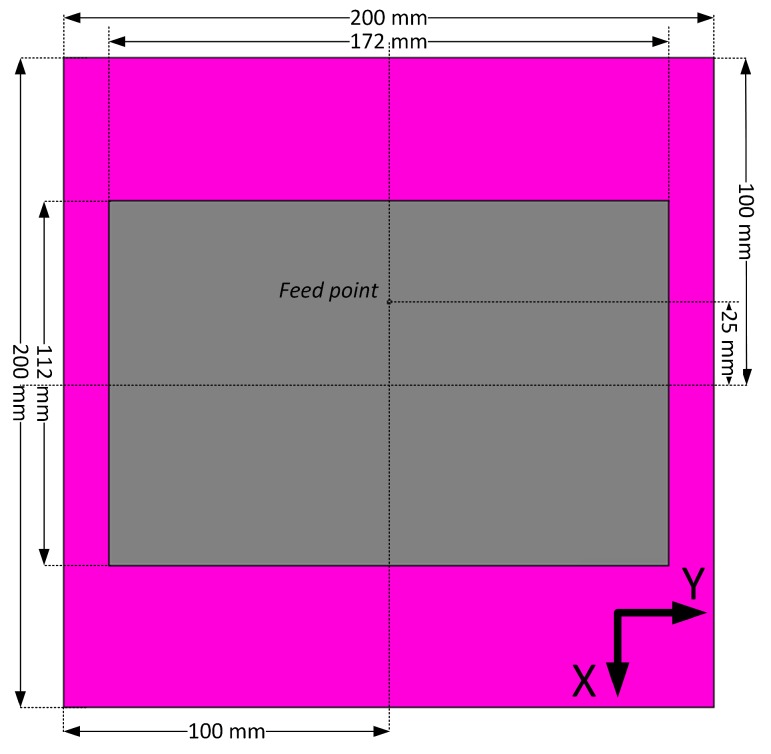
Layout and dimensions of the designed antenna.

Given the geometry of our target application setup, the use of directive antennas is reasonable. The conducted market analyses have shown that no directive 868 MHz band commercial antennas are available. Due to this reason, we designed and prototyped our own linearly-polarized patch antenna, layout, and dimensions, which are illustrated in Figure 6. The reason for us basing our design on the patch antenna was twofold. First, this type of antenna is known to feature higher directivity than the dipoles. At the very same time, these antennas are still sufficiently simple and technological for mass-scale production.

**Figure 7:**
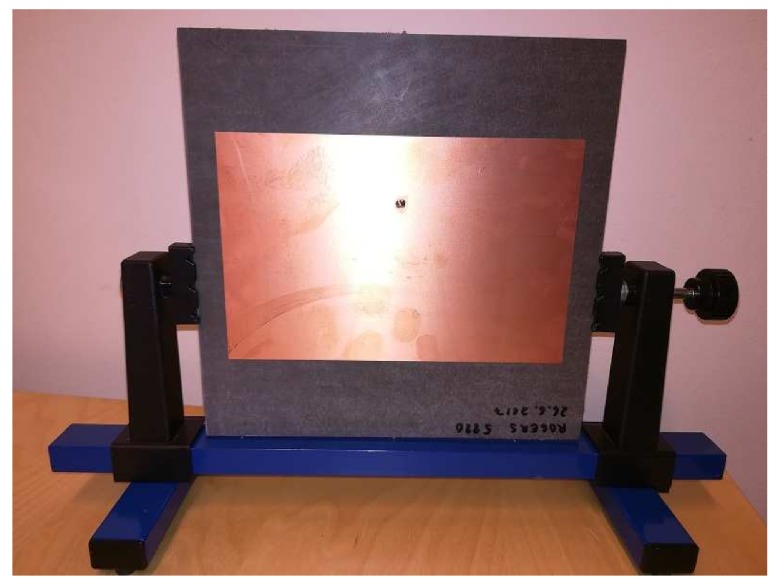
Prototyped antenna for energy transmission.

The designed antenna was first simulated in SW, and then two pieces have been manufactured using a low loss Rogers RT5880 substrate (ϵ=2.20, loss δ=0.0009) with 3.1 mm substrate thickness and a 35 μm copper cladding. The dimensions of the ground plane are 200 × 200 mm^2^ and the dimensions of the radiator are 172 × 112 mm^2^. A large ground plane surface increases the antenna size but also enables a more directive beam and makes the characteristics of the antenna less dependent on mounting location and environment. This makes the antenna better fit our targeted environment. The 50 Ohm coaxial line is used to feed the antenna. One of the manufactured antennas is depicted in Figure 7.

**Figure 8:**
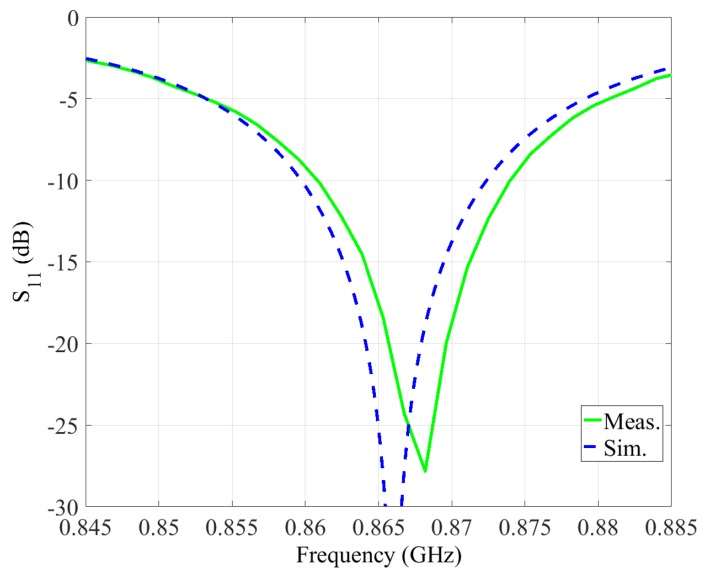
Simulated and measured S11 parameter of the designed antenna.

**Figure 9:**
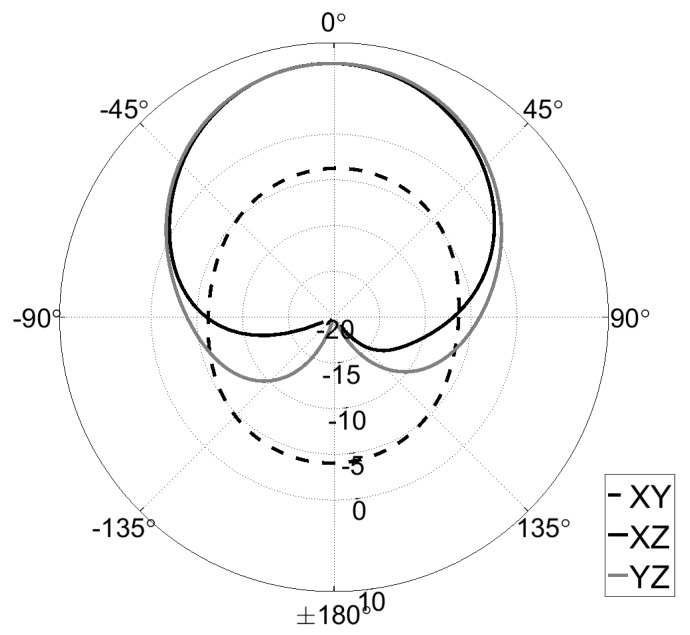
Measured radiation pattern of the designed antenna.

The simulated and measured values of S11 parameter and the radiation pattern of the designed antenna, demonstrating its performance, are depicted in Figure 8 and Figure 9, respectively. The resulting efficiency of the designed antenna, defined as the power radiated over a sphere compared to the power delivered to the antenna port, is on the order of 87–98% for the 860–870 MHz band. The maximum antenna gain is 7.55 dB at 865 MHz, and the width of the beam (according to the 3 dB level) is 75 degrees.

#### 3.4.3. RF–DC Converter

The third key component of the solution is the RF–DC converter. Likewise, for the antenna, there are almost no ready-made commercial RF–DC solutions available today. The only exception to this rule is the P2110B RF energy harvesting chipset [18] from Powercast^®^ (Pittsburgh, PA, USA), the cost of which exceeds 30 EUR (34 USD) for one chip [64]. In larger quantities, the cost may reduce, but still stays rather high. Therefore, we developed our own solution, which is discussed in what follows.

The first challenge with respect to the RF–DC converter design is the selection of the diodes to be used. The components need to be picked mindful of such parameters as the threshold and reverse-breakdown voltage, element parasitics, and harmonics. The former parameter defines the minimum level of the radio signal, from which the circuit could collect the energy (i.e., the “sensitivity” of the circuit), and affects the efficiency of the energy harvesting. The junction resistances and capacitances as well as the package inductances and capacitances influence the efficiency and limit the maximum operating frequency. Finally, the cost and the dimensions of the diodes also play a role, since a single rectifier circuit may employ multiple diodes.

Second, one has to decide the architecture and the number of stages in the rectifying circuitry. Some of the possibilities are reported and discussed, e.g., in Ref. [11]. Importantly, the number of stages needs to be specified based on the expected range of the input radio signal’s power. Specifically, more stages enable collecting the energy from weaker signals, but at the same time introduce some losses which affect the efficiency of the system when harvesting the power from stronger signals. In addition, the more stages increase the dimensions and the price of the system. The former challenge can be addressed by employing a multi-path or switching rectifiers (e.g., the ones proposed in Ref. [65]); nonetheless, this makes the design of a rectifier even more complex and costly.

Finally, the rectifier has to be matched with the antenna to prevent the energy reflections to the environment. Such matching is a non-trivial task due to the nonlinearity of the diode’s characteristics and namely the variation of the diode’s impedance depending on the power and frequency of the input radio signal and needs to account for the specifics of the Printed Circuit Board (PCB) and the manufacturing processes.

**Figure 10:**
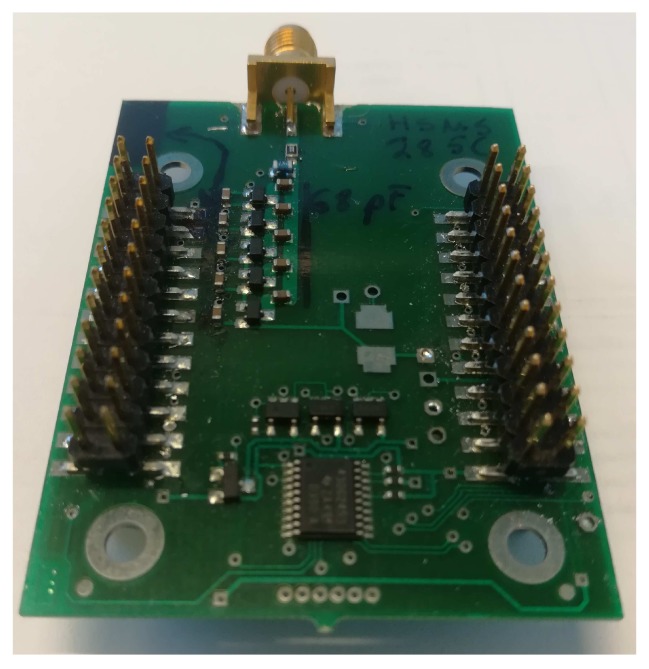
A prototype of energy harvesting printed circuit board.

**Figure 11:**
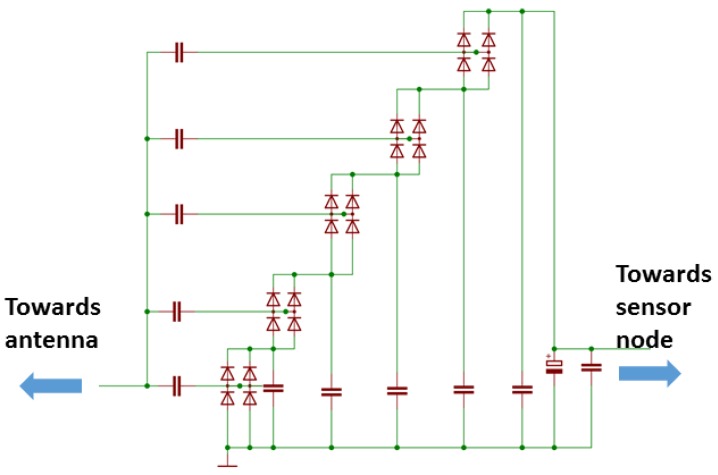
Schematic of the radio frequency to direct current converter board.

For this study, we have designed and produced own energy harvesting circuitry shown in Figure 10. The form factor of the PCB is such that it can be connected to our in-house developed modular IoT HW platform [2,66]. For the board, we used FR4 with 0.8 mm substrate height and 0.18 μm trace thickness. The substrate dielectric is $E_{r}=4.8$. With these parameters, 1.4 mm wide line provides 50 Ohms impedance. We applied a Dickson charge pump built using the Avago HSMS 285C pair serial connected diodes (San Jose, CA, USA), which are optimized for low voltage operation. The schematic of the designed RF–DC converter board is illustrated in Figure 11.

**Figure 12:**
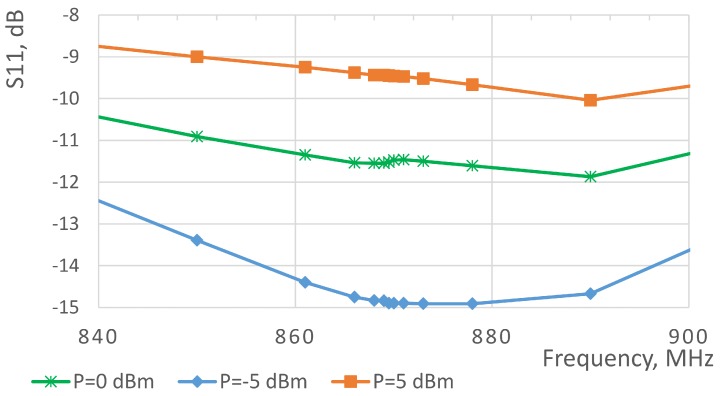
Measured S11 parameter of the radio frequency to direct current converter board for different input power levels.

After assembling the PCB, its matching to the antenna was manually tuned. Namely, we used the Anritsu MS4623B vector network analyzer (Atsugi, Kanagawa, Japan) to estimate the input impedance and used it to calculate the matching components. Furthermore, the matching components were fine-tuned to fight the imperfections and tolerances of the components themselves, as well as to address the effects of manual component assembly. The measured reflection coefficients for the designed board are illustrated in Figure 12. We also measured the efficiencies of the designed RF–DC converter board for the different power levels—the respective results are presented in Table 4. As one can see, the performance is in the same range as that of the state-of-the-art works, discussed in Section 2. In addition, one can see that the designed board demonstrates the peak efficiency for the input signals with units-of-milliWatt power levels, which is well in line with what is needed for our application. Note that the reported measurement results were obtained having the resistive load of 9 kOhm connected to the circuit. The level of the load was selected based on the mean power consumption of the sensor node running our target application. A few iterations of the RF–DC converter design were executed to find the appropriate balance between the efficiency and the level of the output voltage, which needs to exceed the minimum threshold voltage for sensor node’s DC–DC converter.

#### 3.4.4. RF Power Source and Gateway

The two final components of our envisaged system are the wireless power transmitter and a gateway for receiving the reports. We start with the latter. Since we have selected the BLE to be the radio access technology in our test system, this provides us with a sheer diversity of options for the gateway. Specifically, to receive the advertising packets sent by our device, the dedicated BLE transceivers or the mobile devices (i.e., tablets, smartphones or laptops) can be used. For our test case, we have developed two gateway options. The former is a dedicated HW BLE gateway, which monitors and logs the data sent by the WPT sensor for further processing. Another option bases on an Android-based tablet computer, for which we developed a simple application to read the data received from BLE and display them on the screen in real time.

Finally, the strong-enough source of the radio signal to power the designed sensor solution was needed. At first, we attempted to find a commercial solution. The only one available in the market is the TX91501 device [67] from Powercast^®^ (Pittsburgh, PA, USA). Unfortunately, this device did not fit us since it:

- Operates in 915 MHz US ISM band,
- Has a fixed power level of either 1 or 3 Watt,
- Does not enable duty-cycling,
- Does not permit the connection of external antennas.

Therefore, we decided to design the solution matching better our needs. This was done based on our IoT modular HW platform [2,66]. Specifically, we used a node composing a microcontroller and an 868 MHz ISM-band LoRaWAN radio transceiver (the RN2483 from the Microchip (Chandler, AZ, USA) in our case). The transceiver was forced into the test mode, transmitting the radio carrier. The microcontroller can switch the transmission on and off, thus enabling to implement duty-cycling. Since the radio transceiver can transmit with the maximum power of only 14 dBm, we connected to it an external amplifier—the Kalmus 710FC (Souderton, PA, USA). This device amplifies the signal to the level of 27 dBm (500 mW) and applies it to an external antenna—the one we described earlier.

### 3.5. Integration and Evaluation

**Figure 13:**
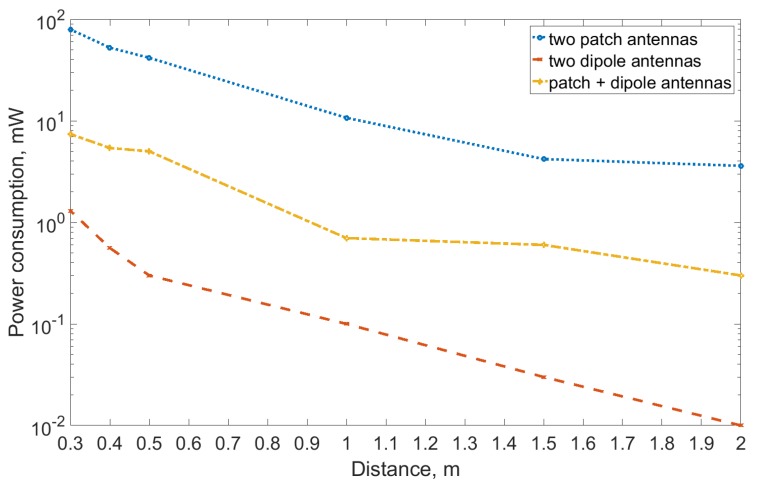
Harvested energy with patch and dipole antennas.

Finally, we integrated all the individual elements of the system. Before mounting them to the target environment, we tested them in a static operation case. For this, we connected the designed antenna to the developed WPT transmitter and enabled the latter. The second designed antenna was connected to the Hewlett-Packard EPM-442A power meter (Palo Alto, CA, USA), which was used to measure the power of the received radio signal. The antennas were pointed towards each other. Note that, for the reference, we repeated the very same experiment using the dipole antenna (Linx Technologies ANT-868-CW-HWR-ccc half wave center-fed antenna (Merlin, OR, USA) with a peak gain of −2.3 dBi) on both ends, and using one patch and one dipole antenna. Figure 13 and Table 5 illustrate the results.

**Figure 14:**
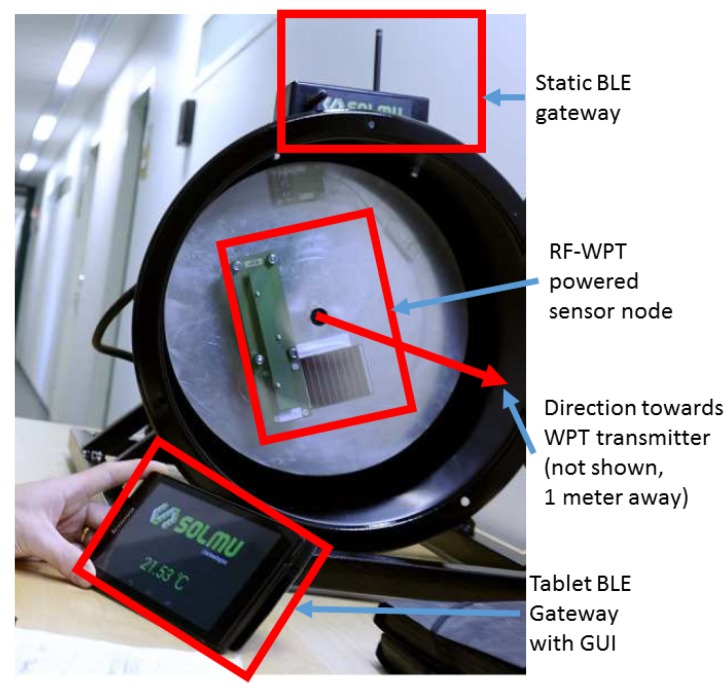
Wireless sensor node application in a rotating environment enabled with wireless power transfer.

The results reveal that the level of the power delivered when using directional antennas exceeded that for omnidirectional ones almost two orders of magnitude. For example, at the one-meter distance, the system equipped with patch antennas delivered 10.7 mW, while the one using dipoles—only 0.10 mW. Given the RF–DC conversion efficiency of 50–60%, the former looked promising. Therefore, we instrumented the complete application by attaching the sensor node and mounting all the components to a rotating stand. This set-up is depicted in Figure 14. Note that the WPT transmitter was placed perpendicular to the axis of shaft’s rotation.

The further experiments with the set-up showed that, at the distance of up to one meter, the WPT transceiver operating with 500 mW transmit power provides sufficient energy for non-intermittent operation of the sensor node. Specifically, the sensor node could sense both the acceleration and the temperature, process the data (e.g., calculate the FFT and estimate the angular speed, and report these data twice per second over BLE advertisements). The results were received by a tablet computer over a BLE link, and can further be injected in a database or forwarded anywhere over the Internet. Note that the antenna used in our experiments was vertically polarized and thus the receiver was not receiving the energy constantly. We also confirmed that longer distances are possible, but then the operation of a sensor node becomes intermittent.

## 4. Conclusions

The research on energy harvesting and transmission continues strong. The idea of a self-powered sensor node is more interesting than ever. At the very same time, the technology is expected to provide new business models through monitoring, tracking and automation capabilities on a large scale. The revolution can be seen everywhere—from connected farms and agriculture to smart cities buildings and homes. Today, the energy harvesting technology enables the gain in the restricted number of applications due to the lack of a universal solution which would provide enough energy to displace batteries and the mains in low power applications. The principal obstacles are the size of the implementation, the high cost and the maturity of the technology.

In the current paper, we have developed a practical IoT application for the industrial rotating environment, powered solely by RF-based wireless power transfer. On this way, we first proposed and discussed the methodology to be utilized when developing such an application. Then, we described in details the complete procedure of developing and evaluating the different components of our solution, and the application as a whole. On the one hand, our results confirm that the proposed development methodology is valid. On the other hand, our results show that, even with the state-of-the-art technology, the practical WPT applications are feasible. This may enable the new and interesting use cases—like sensing in a rotating environment, which we have addressed in this paper.

At the very same time, there are many various challenges associated with the development of WPT-based applications. First, today, the development of an RF-based WPT-based application is still quite a challenging task, requiring diverse expertise. One of the main reasons for this is the fact that the number of the available today commercial components for WPT is scarce and their price is rather high—thus, many system components have to be developed from scratch. In this respect, the recipes contained in this paper may be especially valuable. Second, there is an obvious gap with respect to the WPT-focused frequency spectrum regulations, which introduces some uncertainty also to the process of designing and using the WPT applications. Third, there are still many open research questions regarding the environmental effects (both the effects on nature and health, and the effect of electromagnetism on the machines and materials), which can be potentially associated with WPT. In addition, when done in-band with the traditional communication, the WPT may cause interferences to the latter. Finally, the business prospects of the WPT solutions are still not very clear—does the long-distance WPT where only some percents of the total power consumed by the WPT transmitter are effectively delivered to the receiver make sense? If it does, for which applications? All of these issues still need to be investigated further.

Meanwhile, the efficiency of the design reported in this paper can be further improved in several ways. First, a more directive antenna can be used. Second, the design of the antenna may be changed to make it circularly polarized. Alternatively, the duty-cycling of the WPT transmitter may be synchronized with the rotation of the sensor, thus minimizing the energy losses due to polarization mismatch. Third, some performance improvement can also be achieved by optimizing further the number of the stages in the pump and its matching. Replacing the sensors with the most recently developed ones can also enable some energy savings. Finally, the multi-band RF energy harvesting (e.g., collecting the energy from both bands g3 and h1.7) may also be feasible. We plan to focus on some of these issues in the future.

## Figures and Tables

**Table 1:** Radio frequency to direct current conversion efficiency of selected wireless power transfer solutions (based on Refs. [11,19,20,21,22,23,24,25,26,27,28]).

Peak Efficiency (%)	Input Signal for Peak Efficiency (dBm)	Frequency Band (GHz)	Source
40	−10	0.25–3	[24]
65	0	0.7–1	[25]
10	−22.6	0.906	[29]
26.5	−11.1	0.900	[30]
60	−3	0.915	[31]
78	22	0.915	[21]
60	5	0.915	[22]
80	17	1.0	[27]
37	−25.7	2.450	[32]
60	−5	2.450	[33]
90.6	39	2.450	[34]
74.9	10.1	2.450	[23]
68	5	2.450	[26]
70	5	2.5	[28]
23	−10	5.800	[35]
82	17	5.800	[34]
85.5	37.8	5.800	[19]
60	21.5	10.000	[36]
4.5	−2	25.700	[37]
70	7.7	35.000	[38]
38	20.2	94.000	[20]

**Table 2:** Consumption of the state-of-art commercial sensors.

Sensor Type	Consumption min	Consumption max
**Movement**		
Acclerometers	dozens μW	hundreds μW
Gyroscopes	units mW	dozens mW
**Environmental Conditions**		
Ambient temperature	units μW	hundreds μW
Ambient pressure	dozens μW	dozens μW
Ambient humidity	units μW	hundreds μW
Ambient light (incl. UV)	dozens μW	units mW
Air quality	dozens μW	hundreds mW
Gas presence	hundreds μW	hundreds mW
Dust (optical)	dozens mW	dozens mW
Smoke (optical)	hundreds μW	units mW
**Presence**		
Magnetic/compass	hundreds μW	units mW
Touch	dozens μW	hundreds μW
**Physical Conditions**		
Heart rate	units mW	dozens mW
Gestures (optical)	units mW	dozens mW
**Other**		
Audio	hundreds μW	dozens mW
Video	dozens mW	hundreds mW

**Table 3:** Sub-bands for non-specific short range devices (based on Refs. [60,61]).

Subband [ERC 70-03]	Frequency (MHz)	Effective Radiated Power (mW/dBm)	Duty Cycle (%)
g1	433.050–434.790	≤25/14	≤10
g2	433.050–434.790	≤1/0(−13 dBm/10 kHz)	No restriction
g3	433.040–434.790	≤10/10	No restriction
h1.1–1.3	863.000–870.000	≤25/14	≤0.1
h1.4	868.000–868.600	≤25/14	≤1
h1.5	868.700–869.200	≤25/14	≤0.1
h1.6	869.400–869.650	≤500/27	≤10
h1.7	869.700–870.000	≤5/7	No restriction
h1.7	869.700–870.000	≤25/14	≤1
h2	870.700–873.000	≤25/14	≤1
i	2400.000–2483.500	≤10/10 (EIRP)	No restriction
j	5725.000–5875.000	≤25/14 (EIRP)	No restriction

**Table 4:** Radio frequency to direct current converter efficiency.

Received Power	Power after DC	Max Voltage (V)	Efficiency (%)
(dBm/mW)	Conversion (mW)		
5/10.00	2.04	4.12	20
0/1.00	0.59	2.21	59
−5/0.32	0.16	1.15	51
−10/0.10	0.038	0.56	38

**Table 5:** Harvested energy with patch and dipole type transceiver antennas with 500 mW (27 dBm) transmission power.

Distance (m)	Patch (mW)	Dipole (mW)	Patch + Dipole (mW)
2	3.6	0.01	0.30
1.5	4.2	0.03	0.60
1	10.7	0.10	0.70
0.5	41.7	0.30	5.00
0.4	52.5	0.56	5.40
0.3	79.40	1.30	7.40

## Abbreviations

The following abbreviations are used in this manuscript:

BLE	Bluetooth Low Energy
DC	Direct Current
EIRP	Effective Isotropic Radiated Power
FFT	Fast Fourier Transform
HW	hardware
IEEE	Institute of Electrical and Electronics Engineers
ISM	Industrial, Scientific and Medical
IoT	Internet of Things
LPWAN	Low Power Wide Area Network
LTE	Long Term Evolution
PCB	Printed Circuit Board
RF	Radio Frequency
RFID	Radio Frequency Identification
SW	software
SWIPT	Simultaneous Wireless Information and Power Transfer
WPT	Wireless Power Transfer
WSN	Wireless Sensor Networks
